# Methods for Estimating Item-Score Reliability

**DOI:** 10.1177/0146621618758290

**Published:** 2018-04-09

**Authors:** Eva A. O. Zijlmans, L. Andries van der Ark, Jesper Tijmstra, Klaas Sijtsma

**Affiliations:** 1 Tilburg University, Tilburg, Netherlands; 2 University of Amsterdam, Amsterdam, Netherlands

**Keywords:** correction for attenuation, Guttman’s method λ_6_, item-score reliability, latent class reliability coefficient, method MS

## Abstract

Reliability is usually estimated for a test score, but it can also be estimated for item scores. Item-score reliability can be useful to assess the item’s contribution to the test score’s reliability, for identifying unreliable scores in aberrant item-score patterns in person-fit analysis, and for selecting the most reliable item from a test to use as a single-item measure. Four methods were discussed for estimating item-score reliability: the Molenaar–Sijtsma method (method MS), Guttman’s method $\lambda_{6}$, the latent class reliability coefficient (method LCRC), and the correction for attenuation (method CA). A simulation study was used to compare the methods with respect to median bias, variability (interquartile range [IQR]), and percentage of outliers. The simulation study consisted of six conditions: standard, polytomous items, unequal α parameters, two-dimensional data, long test, and small sample size. Methods MS and CA were the most accurate. Method LCRC showed almost unbiased results, but large variability. Method $\lambda_{6}$ consistently underestimated item-score reliabilty, but showed a smaller IQR than the other methods.

## Introduction

Reliability of measurement is often considered for test scores, but some authors have argued that it may be useful to also consider the reliability of individual items (Ginns & Barrie, 2004; Meijer & Sijtsma, 1995; Meijer, Sijtsma, & Molenaar, 1995; Wanous & Reichers, 1996; Wanous, Reichers, & Hudy, 1997). Just as test-score reliability expresses the repeatability of test scores in a group of people keeping administration conditions equal (Lord & Novick, 1968, p. 65), item-score reliability expresses the repeatability of an item score. Items having low reliability are candidates for removal from the test. Item-score reliability may be useful in person-fit analysis to identify item scores that contain too little reliable information to explain person fit (Meijer & Sijtsma, 1995). Meijer, Molenaar, and Sijtsma (1994) showed that fewer items are needed for identifying misfit when item-score reliability is higher. If items are meant to be used as single-item measurement instruments, their suitability for the job envisaged requires high item-score reliability. Single-item instruments are used in work and organizational psychology for selection and assessing, for example, job satisfaction (Gonzalez-Mulé, Carter, & Mount, 2017; Harter, Schmidt, & Hayes, 2002; Nagy, 2002; Robertson & Kee, 2017; Saari & Judge, 2004; Zapf, Vogt, Seifert, Mertini, & Isic, 1999) and level of burnout (Dolan et al., 2014). Item-score reliability is also used in health research for measuring, for example, quality of life (Stewart, Hays, & Ware, 1988; Yohannes, Willgoss, Dodd, Fatoye, & Webb, 2010) and psychosocial stress (Littman, White, Satia, Bowen, & Kristal, 2006), and one-item measures have been assessed in marketing research for measuring ad and brand attitude (Bergkvist & Rossiter, 2007).

Several authors have proposed methods for estimating item-score reliability. Wanous and Reichers (1996) proposed the correction for attenuation (method CA) for estimating item-score reliability. Method CA correlates an item score and a test score both assumed to measure the same attribute. Google Scholar cited Wanous et al. (1997) 2,400+ times, suggesting method CA is used regularly to estimate item-score reliability. The authors proposed to use method CA for estimating item-score reliability for single-item measures that are used, for example, for measuring job satisfaction (Wanous et al., 1997). Meijer et al. (1995) advocated using the Molenaar-Sijtsma method (method MS; Molenaar & Sijtsma, 1988), which at the time was available only for dichotomous items. In this study, method MS was generalized to polytomous item scores. Two novel methods were also proposed, one based on coefficient $\lambda_{6}$ (Guttman, 1945) denoted as method $\lambda_{6}$, and the other based on the latent class reliability coefficient (Van der Ark, Van der Palm, & Sijtsma, 2011), denoted as method LCRC. This study discusses methods MS, $\lambda_{6}$, LCRC, and CA, each suitable for polytomous item scores, and compared the methods with respect to median bias, variability expressed as interquartile range (IQR), and percentage of outliers. This study also showed that the well-known coefficients α (Cronbach, 1951) and $\lambda_{2}$ (Guttman, 1945) are inappropriate for being used as item-score reliability methods.

Because item-score reliability addresses the repeatability of item scores in a group of people, it provides information different from other item indices. Examples are the corrected item-total correlation (Nunnally, 1978, p. 281), which quantifies how well the item correlates with the sum score on the other items in the test; the item-factor loading (Harman, 1976, p. 15), which quantifies how well the item is associated with a factor score based on the items in the test, and thus corrects for the multidimensionality of total scores; the item scalability (Mokken, 1971, pp. 151-152), which quantifies the relationship between the item and the other items in the test, each item corrected for the influence of its marginal distribution on the relationship; and the item discrimination (e.g., see Baker & Kim, 2004, p. 4), which quantifies how well the item distinguishes people with low and high scores on a latent variable the items have in common. None of these indices addresses repeatability; hence, item-score reliability may be a useful addition to the set of item indices. A study that addresses the formal relationship between the item indices would more precisely inform us about their differences and similarities, but such a theoretical study is absent in the psychometric literature.

Following this study, which focused on the theory of item-score reliability, Zijlmans, Tijmstra, Van der Ark, and Sijtsma (2017) estimated methods MS, $\lambda_{6}$, and CA from several empirical data sets to investigate the methods’ practical usefulness and values that are found in practice and may be expected in other data sets. In addition, the authors estimated four item indices (item-rest correlation, item-factor loading, item scalability, and item discrimination) from the empirical data sets. The values of these four item indices were compared with the values of the item-score reliability methods, to establish the relationship between item-score reliability and the other four item indices.

This article is organized as follows. First, a framework for estimating item-score reliability and three of the item-score reliability methods in the context of this framework are discussed. Second, a simulation study, its results with respect to the methods’ median bias, IQR, and percentage of outliers, and a real-data example are discussed. Methods to use in practical data analysis are recommended.

## A Framework for Item-Score Reliability

The following classical test theory (CTT) definitions (Lord & Novick, 1968, p. 61) were used. Let X be the test score, which is defined as the sum of J item scores, indexed i(i=1,…,J), that is, $X=\sum_{i=1}^{J}X_{i}$. In the population, test score X has variance $\sigma_{X}^{2}$. True score T is the expectation of an individual’s test score across independent repetitions, and represents the mean of the individual’s propensity distribution (Lord & Novick, 1968, pp. 29-30). The deviation of test score X from true score T is the random measurement error, E; that is, E=X−T. Because T and E are unobservable, their variances are also unobservable. Using these definitions, test-score reliability is defined as the proportion of observed-score variance that is true-score variance or, equivalently, one minus the proportion of observed-score variance that is error variance. Mathematically, reliability also equals the product-moment correlation between parallel tests (Lord & Novick, 1968, p. 61), denoted by $\rho_{\mathrm{XX}\prime }$; that is,

$$\rho_{\mathrm{XX}\prime }=\frac{\sigma_{T}^{2}}{\sigma_{X}^{2}}=1-\frac{\sigma_{E}^{2}}{\sigma_{X}^{2}}.$$

Next to notation i, we need j to index items. Notation x and y denote realizations of item scores, and without loss of generality, it is assumed that x,y=0,1,…,m. Let $\pi_{x(i)}=P(X_{i}\geq x)$ be the marginal cumulative probability of obtaining at least score x on item i. It may be noted that $\pi_{0(i)}=1$ by definition. Likewise, let $\pi_{x(i),y(j)}=P(X_{i}\geq x,X_{j}\geq y)$ be the joint cumulative probability of obtaining at least score x on item i and at least score y on item j.

In what follows, it is assumed that index i′ indicates an independent repetition of item i. Let $\pi_{x(i),y(i\prime )}$ denote the joint cumulative probability of obtaining at least score x and at least score y on two independent repetitions, denoted by i and i′, of the same item in the same group of people. Because independent repetitions are unavailable in practice, the joint cumulative probabilities $\pi_{x(i),y(i\prime )}$ have to be estimated from single-administration data.

Molenaar and Sijtsma (1988) showed that reliability (Equation 1) can be written as

$$\rho_{\mathrm{XX}\prime }=\frac{\sum_{i=1}^{J}\sum_{j=1}^{J}\sum_{x=1}^{m}\sum_{y=1}^{m}\left[\pi_{x\left(i\right),y\left(j\right)}-\pi_{x\left(i\right)}\pi_{y\left(j\right)}\right]}{\sigma_{X}^{2}}.$$

Equation 2 can be decomposed into the sum of two ratios:

$$\rho_{\mathrm{XX}\prime }=\frac{\overset{J}{\underset{i\neq j}{\sum \sum }}\sum_{x=1}^{m}\sum_{y=1}^{m}\left[\pi_{x\left(i\right),y\left(j\right)}-\pi_{x\left(i\right)}\pi_{y\left(j\right)}\right]}{\sigma_{X}^{2}}+\frac{\sum_{i=1}^{J}\sum_{x=1}^{m}\sum_{y=1}^{m}\left[\pi_{x\left(i\right),y\left(i\prime \right)}\;-\pi_{x\left(i\right)}\pi_{y\left(i\right)}\right]}{\sigma_{X}^{2}}.$$

Except for the joint cumulative probabilities pertaining to the same item $\pi_{x(i),y(i\prime )}$, all other terms in Equation 3 are observable and can be estimated from the sample. Van der Ark et al. (2011) showed that for test score X, the single-administration reliability methods α, $\lambda_{2}$, MS, and LCRC only differ with respect to the estimation of $\pi_{x(i),y(i\prime )}$.

To define item-score reliability, Equation 3 can be adapted to accommodate only one item; the first ratio and the first summation sign in the second ratio disappear, and item-score reliability $\rho_{\mathrm{ii}\prime }\;$is defined as

$$\rho_{\mathrm{ii}\prime }\;=\frac{\sum_{x=1}^{m}\sum_{y=1}^{m}\left[\pi_{x\left(i\right),y\left(i\prime \right)}\;-\pi_{x\left(i\right)}\pi_{y\left(i\right)}\right]}{\sigma_{X_{i}}^{2}}=\frac{\sigma_{T_{i}}^{2}}{\sigma_{X_{i}}^{2}}.$$

## Methods for Approximating Item-Score Reliability

Three of the four methods that were investigated, methods MS, $\lambda_{6}$, and LCRC, use different approximations to the unobservable joint cumulative probability $\pi_{x(i),y(i\prime )}$, and fit into the same reliability framework. Two other well-known methods that fit into this framework, Cronbach’s α and Guttman’s $\lambda_{2}$, cannot be used to estimate item-score reliability (see Appendix). The fourth method, CA, uses a different approach to estimating item-score reliability and conceptually stands apart from the other three methods. All four methods estimate Equation 4, which contains two unknowns - in addition to $\rho_{\mathrm{ii}\prime }$ bivariate proportion $\pi_{x(i),y(i\prime )}$ (middle) and variance $\sigma_{T_{i}}^{2}$ (right) - and thus cannot be estimated directly from the data.

### Method MS

Method MS uses the available marginal cumulative probabilities to approximate $\pi_{x(i),y(i\prime )}$. The method is based on the item response model known as the double monotonicity model (Mokken, 1971; Sijtsma & Molenaar, 2002). This model is based on the assumptions of a unidimensional latent variable; independent item scores conditional on the latent variable, which is known as local independence; response functions that are monotone nondecreasing in the latent variable; and nonintersection of the response functions of different items. The double monotonicity model implies that the observable bivariate proportions $\pi_{x(i),y(j)}$ collected in the P(++) matrix are nondecreasing in the rows and the columns (Sijtsma & Molenaar, 2002, pp. 104-105). The structure of the P(++) matrix using an artificial example is illustrated.

For four items, each having three ordered item scores, Table 1 shows the marginal cumulative probabilities. First, ignoring the uninformative $\pi_{0i}=1$, the authors assume that probabilities can be strictly ordered, and order the eight remaining marginal cumulative probabilities in this example from small to large:

$$\pi_{2(2)}<\pi_{2(1)}<\pi_{2(4)}<\pi_{2(3)}<\pi_{1(4)}<\pi_{1(3)}<\pi_{1(2)}<\pi_{1(1)}.$$

**Table 1.** Marginal Cumulative Probabilities for Four Artificial Items With Three Ordered Item Scores.

	Item			
	1	2	3	4
$\pi_{0(i)}$	1.00	1.00	1.00	1.00
$\pi_{1(i)}$	.97	.94	.93	.86
$\pi_{2(i)}$	.53	.32	.85	.72

Van der Ark (2010) discussed the case in which Equation 5 contains ties. Second, the P(++) matrix is defined, which has order Jm×Jm and contains the joint cumulative probabilities. The rows and columns are ordered reflecting the ordering of the marginal cumulative probabilities, which are arranged from small to large along the matrix’ marginals; see Table 2. The ordering of the marginal cumulative probabilities determines where each of the joint cumulative probabilities is located in the matrix. For example, the entry in cell (4,7) is $\pi_{2(3),1(2)}$, which equals .81. Mokken (1971, pp. 132-133) proved that the double monotonicity model implies that the rows and the columns in the P(++) matrix are nondecreasing. This is the property on which method MS rests. In Table 2, entry NA (i.e., not available) refers to the joint cumulative probabilities of the same item, which are unobservable. For example, in cell (5,3), the proportion $\pi_{1(4),2(4\prime )}$ is NA and hence cannot be estimated numerically.

**Table 2.** P(++) Matrix With Joint Cumulative Probabilities $\pi_{x(i),y(j)}$ and Marginal Cumulative Probabilities $\pi_{x(i)}$.

		$\pi_{2(2)}$	$\pi_{2(1)}$	$\pi_{2(4)}$	$\pi_{2(3)}$	$\pi_{1(4)}$	$\pi_{1(3)}$	$\pi_{1(2)}$	$\pi_{1(1)}$
		.32	.53	.72	.85	.86	.93	.94	.97
$\pi_{2(2)}$	.32	NA	.20	.27	.29	.30	.31	NA	.32
$\pi_{2(1)}$	.53	.20	NA	.41	.47	.48	.50	.51	NA
$\pi_{2(4)}$	.72	.27	.41	NA	.64	NA	.68	.68	.70
$\pi_{2(3)}$	.85	.29	.47	.64	NA	.76	NA	.81	.84
$\pi_{1(4)}$	.86	.30	.48	NA	.76	NA	.81	.81	.84
$\pi_{1(3)}$	.93	.31	.50	.68	NA	.81	NA	.88	.91
$\pi_{1(2)}$	.94	NA	.51	.68	.81	.81	.88	NA	.91
$\pi_{1(1)}$	.97	.32	NA	.70	.84	.84	.91	.91	NA

*Note.* NA = not available.

Method MS uses the adjacent, observable joint cumulative probabilities of different items to estimate the unobservable joint cumulative probabilities $\pi_{x(i),y(i\prime )}$ by means of eight approximation methods (Molenaar & Sijtsma, 1988). For test scores, Molenaar and Sijtsma (1988) explained that method MS attempts to approximate the item response functions of an item and for this purpose uses adjacent items, because when item response functions do not intersect, adjacent functions are more similar to the target item response function, thus approximating repetitions of the same item, than item response functions further away. When an adjacent probability is unavailable, for example, in the first and last rows and the first and last columns in Table 2, only the available estimators are used. For example, $\pi_{1(1),2(1\prime )}$ in cell (8,2) does not have lower neighbors. Hence, only the proportions .32, cell (8,1); .51, cell (7,2); and .70, cell (8,3) are available for approximating $\pi_{1(1),2(1\prime )}$. For further details, see Molenaar and Sijtsma (1988) and Van der Ark (2010).

Hence, following Molenaar and Sijtsma (1988), the joint cumulative probability $\pi_{x(i),y(i\prime )}$ is approximated by the mean of at most eight approximations resulting in ${\tilde{\pi }}_{x(i),y(i\prime )}^{\mathrm{MS}}$. When the double monotonicity model does not hold, item response functions adjacent to the target item response function may intersect and not approximate the target very well, so that ${\tilde{\pi }}_{x(i),y(i\prime )}^{\mathrm{MS}}$ may be a poor approximation of $\pi_{x(i),y(i\prime )}$. The approximation of $\pi_{x(i),y(i\prime )}$ by method MS is used in Equation 4 to estimate the item-score reliability.

Method MS is equal to item-score reliability $\rho_{\mathrm{ii}\prime }\;$ when $\sum_{x}\sum_{y}\pi_{x(i)y(i\prime )}=\sum_{x}\sum_{y}{\tilde{\pi }}_{x(i)y(i\prime )}^{\mathrm{MS}}$. A sufficient condition is that all the entries in the P(++) matrix are equal; equality of entries requires item response functions that coincide. Further study of this topic is beyond the scope of this article but should be taken up in future research.

### Method $\lambda_{6}$

An item-score reliability method based on Guttman’s $\lambda_{6}$ (Guttman, 1945) can be derived as follows. Let $\epsilon_{i}^{2}$ denote the variance of the estimation or residual error of the multiple regression of item score $X_{i}$ on the remaining J−1 item scores, and determine $\epsilon_{i}^{2}$ for each of the J items. Guttman’s $\lambda_{6}$ is defined as

$$\lambda_{6}=1-\frac{\sum_{i=1}^{J}\epsilon_{i}^{2}}{\sigma_{X}^{2}}.$$

It may be noted that Equation 6 resembles the right-hand side of Equation 1. Let $\Sigma_{\mathrm{ii}}$ denote the (J−1)×(J−1) inter-item variance–covariance matrix for (J−1) items except item i. Let $\sigma_{i}$ be a (J−1)×1 vector containing the covariances of item i with the other (J−1) items. Jackson and Agunwamba (1977) showed that the variance of the estimation error equals

$$\epsilon_{i}^{2}=\sigma_{X_{i}}^{2}-\sigma \prime_{i}{\left(\Sigma_{\mathrm{ii}}\right)}^{-1}\sigma_{i}.$$

When estimating the reliability of an item score, Equation 6 can be adapted to

$$\lambda_{6_{i}}=1-\frac{\sigma_{X_{i}}^{2}-\sigma \prime_{i}{\left(\Sigma_{\mathrm{ii}}\right)}^{-1}\sigma_{i}}{\sigma_{X_{i}}^{2}}=\frac{\sigma \prime_{i}{\left(\Sigma_{\mathrm{ii}}\right)}^{-1}\sigma_{i}}{\sigma_{X_{i}}^{2}}.$$

It can be shown that method $\lambda_{6}$ fits into the framework of Equation 4. Let ${\tilde{\pi }}_{x(i),y(i\prime )}^{\lambda_{6}}$ be an approximation of $\pi_{x(i),y(i\prime )}$ based on observable proportions, such that replacing $\pi_{x(i),y(i\prime )}$ in the right-hand side of Equation 4 by ${\tilde{\pi }}_{x(i),y(i\prime )}^{\lambda_{6}}$ results in $\lambda_{6_{i}}$. Hence,

$$\lambda_{6_{i}}=\frac{\sum_{x=1}^{m}\sum_{y=1}^{m}\left[{\tilde{\pi }}_{x(i),y(i\prime )}^{\lambda_{6}}-\pi_{x(i)}\pi_{y(i)}\right]}{\sigma_{X_{i}}^{2}}.$$

Equating Equation 8 and 9 shows that

$$\begin{array}{c} \frac{\sigma \prime_{i}{\left(\Sigma_{\mathrm{ii}}\right)}^{-1}\sigma_{i}}{\sigma_{X_{i}}^{2}}=\frac{\sum_{x=1}^{m}\sum_{y=1}^{m}\left[{\tilde{\pi }}_{x\left(i\right),y\left(i\prime \right)}^{\lambda_{6}}-\pi_{x\left(i\right)}\pi_{y\left(i\right)}\right]}{\sigma_{X_{i}}^{2}}\Leftrightarrow \\ \frac{\sigma \prime_{i}{\left(\Sigma_{\mathrm{ii}}\right)}^{-1}\sigma_{i}}{m^{2}}={\tilde{\pi }}_{x\left(i\right),y\left(i\prime \right)}^{\lambda_{6}}-\pi_{x\left(i\right)}\pi_{y\left(i\right)}\Leftrightarrow \\ {\tilde{\pi }}_{x\left(i\right),y\left(i\prime \right)}^{\lambda_{6}}=\frac{\sigma \prime_{i}{\left(\Sigma_{\mathrm{ii}}\right)}^{-1}\sigma_{i}}{m^{2}}+\pi_{x\left(i\right)}\pi_{y\left(i\right)}. \end{array}$$

Inserting ${\tilde{\pi }}_{x(i),y(i\prime )}^{\lambda_{6}}$ in Equation 4 yields method $\lambda_{6}$ for item-score reliability. Replacing parameters by sample statistics produces an estimate.

Preliminary computations suggest that only highly contrived conditions produce the equality $\sigma_{T_{i}}^{2}=\sigma \prime_{i}(\Sigma_{\mathrm{ii}}{)}^{-1}\sigma_{i}$ in Equation 8, but conditions more representative for what one may find with real data produce negative item true score variance, also known as Heywood cases. Because this work is premature, the authors tentatively conjecture that in practice, method $\lambda_{6}$ is a strict lower bound to the item-score reliability, a result that is consistent with simulation results discussed elsewhere (e.g., Oosterwijk, Van der Ark, & Sijtsma, 2017).

### Method LCRC

Method LCRC is based on the unconstrained latent class model (LCM; Hagenaars & McCutcheon, 2002; Lazarsfeld, 1950; McCutcheon, 1987). The LCM assumes local independence, meaning that item scores are independent given class membership. Two different probabilities are important, which are the latent class probabilities that provide the probability to be in a particular latent class k(k=1,…,K), and the latent response probabilities that provide the probability of a particular item score given class membership. For local independence given a discrete latent variable ξ with K classes, the unconstrained LCM is defined as

$$P\left(X_{1}=x_{1},...,X_{J}=x_{J}\right)=\sum_{k=1}^{K}P\left(\xi =k\right)\overset{J}{\underset{j=1}{\Pi }}P\left(X_{i}=x_{i}|\xi =k\right).$$

The LCM (Equation 11) decomposes the joint probability distribution of the J item scores for the sum across K latent classes of the product of the probability to be in class k and the conditional probability of a particular item score $X_{i}$. Let ${\tilde{\pi }}_{x(i),y(i\prime )}^{\mathrm{LCRC}}$ be the approximation of $\pi_{x(i),y(i\prime )}$ using the parameters of the unconstrained LCM at the right-hand side of Equation 11, such that

$${\tilde{\pi }}_{x(i),y(i\prime )}^{\mathrm{LCRC}}=\sum_{u=x}^{m}\sum_{v=y}^{m}\sum_{k=1}^{K}P\left(\xi =k\right)P\left(X_{i}=u|\xi =k\right)P\left(X_{i}=v|\xi =k\right).$$

Approximation ${\tilde{\pi }}_{x(i),y(i\prime )}^{\mathrm{LCRC}}$ can be inserted in Equation 4 to obtain method LCRC. After insertion of sample statistics, an estimate of method LCRC is obtained.

Method LCRC equals $\rho_{\mathrm{ii}}\prime$ if $\pi_{x(i),y(i\prime )}$ (Equation 4) equals ${\tilde{\pi }}_{x(i),y(i\prime )}^{\mathrm{LCRC}}$ (Equation 12), hence $\pi_{x(i),y(i\prime )}\;=\sum_{u=x}^{m}\sum_{v=y}^{m}\sum_{k=1}^{K}P(\xi =k)P(X_{i}=u|\xi =k)P(X_{i}=v|\xi =k)$. A sufficient condition for method LCRC to equal $\rho_{\mathrm{ii}}\prime$ is that K has been correctly selected and all estimated parameters P(ξ=k) and $P(X_{i}=x|\xi =k)$ equal the population parameters. This condition is unlikely to be true in practice. In samples, LCRC may either underestimate or overestimate $\rho_{\mathrm{ii}}\prime$.

### Method CA

The CA (Lord & Novick, 1968, pp. 69-70; Nunnally & Bernstein, 1994, p. 257; Spearman, 1904) can be used for estimating item-score reliability (Wanous & Reichers, 1996). Let Y be a random variable, which preferably measures the same attribute as item score $X_{i}$ but does not include $X_{i}$. Likely candidates for Y are the rest score $R_{\left(i\right)}=X-X_{i}$ or the test score on another, independent test that does not include item score $X_{i}$ but measures the same attribute. Let $\rho_{T_{X_{i}}T_{Y}}$ be the correlation between true scores $T_{X_{i}}$ and $T_{Y}$, let $\rho_{X_{i}Y}$ be the correlation between $X_{i}$ and Y, let $\rho_{\mathrm{ii}}\prime$ be the item-score reliability of $X_{i}$, and let $\rho_{\mathrm{YY}}\prime$ be the reliability of Y. Then, method CA equals

$$\rho_{T_{X_{i}}T_{Y}}=\frac{\rho_{X_{i}Y}}{\sqrt{\rho_{\mathrm{ii}}\prime \;}\cdot \sqrt{\rho_{\mathrm{YY}}^{\prime }}}.$$

It follows from Equation 13 that the item-score reliability equals

$$\rho_{\mathrm{ii}}\prime ={\left(\frac{\rho_{X_{i}Y}}{\rho_{T_{X_{i}}T_{Y}}\sqrt{\rho_{\mathrm{YY}}^{\prime }}}\right)}^{2}=\frac{\rho_{X_{i}Y}^{2}}{\rho_{T_{X_{i}}T_{Y}}^{2}\rho_{\mathrm{YY}}^{\prime }}.$$

Let ${\tilde{\rho }}_{\mathrm{ii}}\prime^{\mathrm{CA}}$ denote the item-score reliability estimated by method CA. Method CA is based on two assumptions. First, true scores $T_{X_{i}}$ and $T_{Y}$ correlate perfectly; that is, $\rho_{T_{X_{i}}T_{Y}}=1$, reflecting that $T_{X_{i}}$ and $T_{Y}$ measure the same attribute. Second, $\rho_{\mathrm{YY}}\prime$ equals the population reliability. Because many researchers use coefficient alpha ($\mathrm{alph}a_{Y}$) to approximate $\rho_{\mathrm{YY}}\prime$, in practice, it is assumed that $\mathrm{alph}a_{Y}=\rho_{\mathrm{YY}}\prime$. Using these two assumptions, Equation 14 reduces to

$${\tilde{\rho }}_{\mathrm{ii}}\prime^{\mathrm{CA}}=\frac{\rho_{X_{i}Y}^{2}}{\mathrm{alph}a_{Y}}.$$

Comparing ${\tilde{\rho }}_{\mathrm{ii}}\prime^{\mathrm{CA}}$ and $\rho_{\mathrm{ii}}\prime$, one may notice that ${\tilde{\rho }}_{\mathrm{ii}}\prime^{\mathrm{CA}}=\rho_{\mathrm{ii}}\prime$, if the denominators in Equations 15 and 14 are equal, that is, if $\mathrm{alph}a_{Y}=\rho_{T_{X_{i}}T_{Y}}^{2}\rho_{\mathrm{YY}}\prime$. When does this happen? Assume that $Y=R_{\left(i\right)}$. Then, if the J−1 items on which Y is based are essentially τ-equivalent, meaning that $T_{X_{i}}=T_{Y}+b_{\mathrm{iY}}$ (Lord & Novick, 1968, p. 50), then $\mathrm{alph}a_{Y}=\rho_{\mathrm{YY}}\prime$. This results in $\rho_{\mathrm{YY}}\prime =\rho_{T_{X_{i}}T_{Y}}^{2}\rho_{\mathrm{YY}}\prime$, implying that $\rho_{T_{X_{i}}T_{Y}}^{2}=1$, hence $\rho_{T_{X_{i}}T_{Y}}=1$, and this is true if $T_{X_{i}}$ and $T_{Y}$ are linearly related: $T_{X_{i}}=a_{\mathrm{iY}}T_{Y}+b_{\mathrm{iY}}$. Because it is already assumed that items are essentially τ-equivalent and because the linear relation has to be true for all J items, $b_{i}=0$ for all i and ${\tilde{\rho }}_{\mathrm{ii}}\prime^{\mathrm{CA}}=\rho_{\mathrm{ii}}\prime$ if all items are essentially τ-equivalent. Further study of the relation between ${\tilde{\rho }}_{\mathrm{ii}}\prime^{\mathrm{CA}}$ and $\rho_{\mathrm{ii}}\prime \;$ is beyond the scope of this article, and is referred to future research.

## Simulation Study

A simulation study was performed to compare median bias, IQR, and percentage of outliers produced by item-score reliability methods MS, $\lambda_{6}$, LCRC, and CA. Joint cumulative probability $\pi_{x(i),y(i\prime )}$ was estimated using methods MS, $\lambda_{6}$, and LCRC. For these three methods, the estimates of the joint cumulative probabilities $\pi_{x(i),y(i\prime )}$ were inserted in Equation 4 to estimate the item-score reliability. For method CA, Equation 15 was used.

### Method

Dichotomous or polytomous item scores were generated using the multidimensional graded response model (De Ayala, 1994). Let $\theta =(\theta_{1},\ldots ,\theta_{Q})$ be the Q-dimensional latent variable vector, which has a Q-variate standard normal distribution. Let $\alpha_{\mathrm{iq}}$ be the discrimination parameter of item i relative to latent variable q, and let $\delta_{\mathrm{ix}}$ be the location parameter for category x(x=1,2,…,m) of item i. The multidimensional graded response model (De Ayala, 1994) is defined as

$$P\left(X_{i}\geq x|\theta \right)=\frac{\exp\left[\sum_{q=1}^{Q}\alpha_{\mathrm{iq}}\left(\theta_{q}-\delta_{\mathrm{ix}}\right)\right]}{1+\exp\left[\sum_{q=1}^{Q}\alpha_{\mathrm{iq}}\left(\theta_{q}-\delta_{\mathrm{ix}}\right)\right]}.$$

The design for the simulation study was based on the design used by Van der Ark et al. (2011) for studying test score reliability. A standard condition was defined for six dichotomous items (J=6,m+1=2), one dimension (Q=1), equal discrimination parameters ($\alpha_{\mathrm{iq}}=1$ for all i and q) and equidistantly spaced location parameters $\delta_{\mathrm{ix}}$ ranging from −1.5 to 1.5 (Table 3), and sample size N=1,000. The other conditions differed from the standard condition with respect to one design factor. Test length, sample size, and item-score format were considered extensions of the standard condition, and discrimination parameters and dimensionality were considered deviations, possibly affecting methods the most.

**Table 3.** Item Parameters of the Multidimensional Graded Response Model for the Simulation Design.

Item	Design											
	Standard		Polytomous					Unequal α		Two dimensions		
	$\alpha_{j}$	$\delta_{j}$	$\alpha_{j}$	$\delta_{j1}$	$\delta_{j2}$	$\delta_{j3}$	$\delta_{j4}$	$\alpha_{j}$	$\delta_{j}$	$\alpha_{j1}$	$\alpha_{j2}$	$\delta_{j}$
1	1	−1.5	1	−3	−2	−1	0	0.5	−1.5	1	0	−1.5
2	1	−0.9	1	−2.4	−1.4	−0.4	0.6	2	−0.9	0	1	−0.9
3	1	−0.3	1	−1.8	−0.8	0.2	1.2	0.5	−0.3	1	0	−0.3
4	1	0.3	1	−1.2	−0.2	0.8	1.8	2	0.3	0	1	0.3
5	1	0.9	1	−0.6	0.4	1.4	2.4	0.5	0.9	1	0	0.9
6	1	1.5	1	0	1	2	3	2	1.5	0	1	1.5

*Note.*α = item discrimination, δ = item location.

*Test length* (J): The test consisted of 18 items (J=18). For this condition, the six items from the standard condition were copied twice.
*Sample size* (N): The sample size was small (N=200).
*Item-score format* (m+1): The J items were polytomous (m+1=5).
*Discrimination parameters* (α): Discrimination parameters differed across items (α=.5 or 2). This constituted a violation of the assumption of nonintersecting item response functions needed for method MS.
*Dimensionality* (Q): The items were two-dimensional (Q=2) with latent variables correlating .5. The location parameters alternated between the two dimensions. This condition is more realistic than the condition chosen in Van der Ark et al. (2011), representing two subscale scores that are combined into an overall measure, whereas Van der Ark et al. (2011) used orthogonal dimensions.

Van der Ark et al. (2011) found that item format and sample size did not affect bias of test score reliability, but these factors were included in this study to find out whether results for individual items were similar to results for test scores.

Data sets were generated as follows. For every replication, *N* latent variable vectors, $\theta_{1},\ldots ,\theta_{N}$, were randomly drawn from the θ distribution. For each set of latent variable scores, for each item, the *m* cumulative response probabilities were computed using Equation 16. Using the *m* cumulative response probabilities, item scores were drawn from the multinomial distribution. In each condition, 1,000 data sets were drawn.

Population item-score reliability $\rho_{\mathrm{ii}}\prime$ was approximated by generating item scores for 1 million simulees (i.e., sets of item scores). For each item, the variance based on the **θ**s of the 1 million simulees was divided by the variance of the item score $X_{i}$ to obtain the population item-score reliability. It was found that $.05\leq \rho_{\mathrm{ii}}\prime \leq .41$.

Let $s_{r}$ be the estimate of $\rho_{\mathrm{ii}}\prime \;$ in replication r(r=1,…,R) by means of methods MS, $\lambda_{6}$, and CA. For each method, difference $\left(s_{r}-\rho_{\mathrm{ii}}\prime \right)$ is displayed in boxplots. For each item-score reliability method, median bias, IQR, and percentage of outliers were recorded. An overall measure reflecting estimation quality based on the three quantities was not available, and in cases were a qualification of a method’s estimation quality was needed, the authors indicated how the median bias, IQR, and percentage of outliers were weighted. The computations were done using R (R Core Team, 2015). The code is available via https://osf.io/e83tp/. For the computation of method MS, the package mokken was used (Van der Ark, 2007, 2012). For the computation of the LCM used for estimating method LCRC, the package poLCA was used (Linzer & Lewis, 2011).

### Results

**Figure 1.**
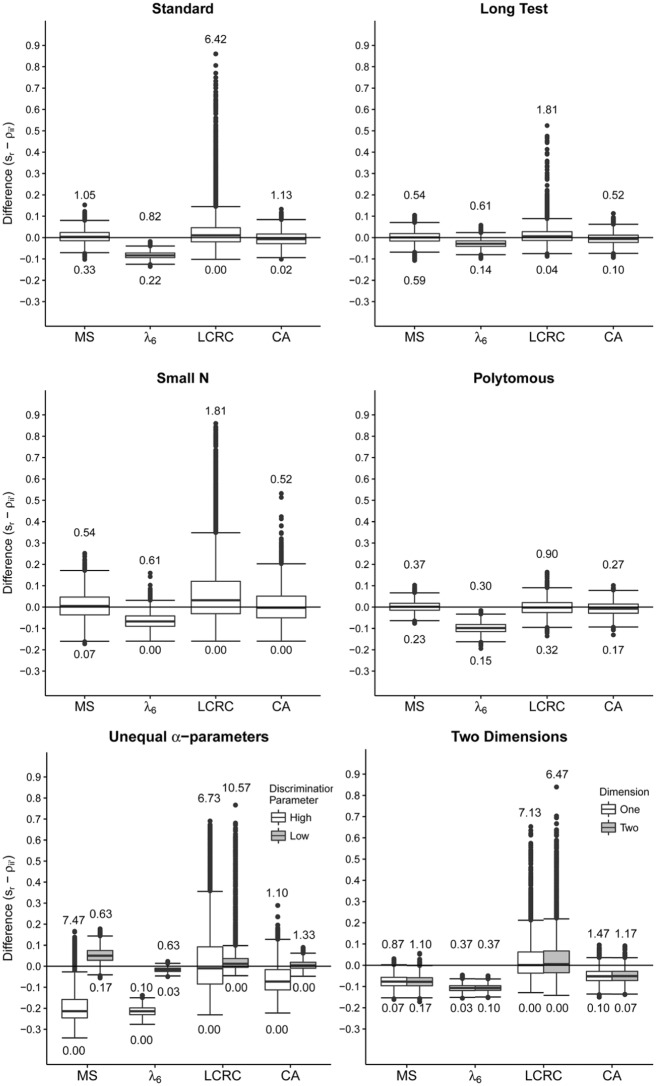
Difference $\left(s_{r}-\rho_{\mathrm{ii}\prime }\right)$, where $s_{r}$ represents an estimate of methods MS, $\lambda_{6}$, LCRC, and CA, for six different conditions (see Table 3 for the specifications of the conditions). *Note.* The bold horizontal line represents the median bias. The numbers in the boxplots represent the percentage outliers in that condition. MS = Molenaar–Sijtsma method; $\lambda_{6}$ = Guttman’s method $\lambda_{6}$; LCRC = latent class reliability coefficient; CA = correction for attenuation.

For each condition, Figure 1 shows the boxplots for the difference $\left(s_{r}-\rho_{\mathrm{ii}}\right)$. In general, differences across items in the same experimental condition were negligible; hence, the results were aggregated not only across replications but also across the items in a condition, so that each condition contained J×1000 estimated item-score reliabilities. The bold horizontal line in each boxplot represents median bias. The dots outside the whiskers are outliers, defined as values that lie beyond 1.5 times the IQR measured from the whiskers of the first and the third quartile. For unequal αs and for Q=2, results are presented separately for high and low αs and for each θ, respectively.

In the standard condition (Figure 1), median bias for methods MS, LCRC, and CA was close to 0. For method LCRC, 6.4% of the difference $\left(s_{r}-\rho_{\mathrm{ii}}\prime \right)$ qualified as an outlier. Hence, compared with methods MS and CA, method LCRC had a large IQR. Method $\lambda_{6}$ consistently underestimated item-score reliability. In the long-test condition (Figure 1), for all methods, the IQR was smaller than in the standard condition. For the small-N condition (Figure 1), for all methods, IQR was a little greater than in the standard condition. In the polytomous item condition (Figure 1), median bias and IQR results were comparable with results in the standard condition, but method LCRC showed fewer outliers (i.e., 1.2%).

Results for high-discrimination items and low-discrimination items can be found in Figure 1, unequal α-parameters condition panel. Median bias was smaller for low-discrimination items. For both high and low-discimination items, method LCRC produced median bias close to 0. Compared with the standard condition, IQR was greater for high-discrimination items and the percentage of outliers was higher for both high- and low-discrimination items. For high-discrimination items, methods MS, $\lambda_{6}$, and CA showed greater negative median bias than for low-discrimination items. For low-discrimination items, method MS had a small positive bias and for methods $\lambda_{6}$ and CA, the results were similar to the standard condition. For the two-dimensional data condition (Figure 1), methods MS and CA produced larger median bias compared with the standard condition. Methods LCRC and CA also produced larger IQR than in the standard condition. Method $\lambda_{6}$ showed smaller IQR than in the standard condition.

A simulation study performed for six items with equidistantly spaced location parameters ranging from −2.5 to 2.5 showed that the number of outliers was larger for all methods, ranging from 0% to 9.6%. This result was also found when the items having the highest and lowest discrimination parameter were omitted.

Depending on the starting values, the expectation maximization (EM) algorithm estimating the parameters of the LCM may find a local optimum rather than the global optimum of the loglikelihood. Therefore, for each item-score reliability coefficient, the LCM was estimated 25 times using different starting values. The best-fitting LCM was used to compute the item-score reliability coefficient. This produced the same results, and left the former conclusion unchanged.

## Real-Data Example

A real-data set illustrated the most promising item-score reliability methods. Because method LCRC had large IQR and a high percentages of outliers and because results were better and similar for the other three methods, methods MS, $\lambda_{6}$, and CA were selected as the three most promising methods. The data set (N=425) consisted of 0/1 scores on 12 dichotomous items measuring transitive reasoning (Verweij, Sijtsma, & Koops, 1999). The corrected item-total correlation, the item-factor loading based on a confirmatory factor model, the item-scalability coefficient (denoted $H_{i}$; Mokken, 1971, pp. 151-152), and the item-discrimination parameter (based on a two-parameter logistic model) were also estimated. The latter four measures provide an indication of item quality from different perspectives, and use different rules of thumb for interpretation. De Groot and Van Naerssen (1969, p. 351) suggested .3 to .4 as minimally acceptable corrected item-total correlations for maximum-performance tests. For the item-factor loading, values of .3 to .4 are most commonly recommended (Gorsuch, 1983, p. 210; Nunnally, 1978, pp. 422-423; Tabachnick & Fidell, 2007, p. 649). Sijtsma and Molenaar (2002, p. 36) suggested to only accept items having $H_{i}\geq .3$ in a scale. Finally, Baker (2001, p. 34) recommended a lower bound of 0.65 for item discrimination.

Using these rules of thumb yielded the following results (Table 4). Only Item 3 met the rules of thumb value for the four item indices. Item 3 also had the highest estimated item-score reliability, exceeding .3 for all three methods. Items 2, 4, 7, and 12 did not meet the rules of thumb of any of the item indices. These items had the lowest item-score reliability not exceeding .3 for any method.

**Table 4.** Estimated Item Indices for the Transitive Reasoning Data Set.

Item	Item-score reliability				Item indices			
	Item *M*	Method MS	Method $\lambda_{6}$	Method CA	Item-rest correlation	Item-factor loading	Item scalability	Item discrimination
$X_{1}$	0.97	**0.36**	0.28	0.21	0.26	**0.85**	0.28	**2.69**
$X_{2}$	0.81	0.01	0.13	0.05	0.13	−0.04	0.08	−0.05
$X_{3}$	0.97	**0.47**	**0.30**	**0.35**	**0.33**	**0.88**	**0.40**	**3.16**
$X_{4}$	0.78	0.05	0.13	0.02	0.08	−0.10	0.05	−0.20
$X_{5}$	0.84	0.18	0.23	**0.31**	0.29	**0.73**	0.18	**1.94**
$X_{6}$	0.94	**0.32**	0.20	0.17	0.23	**0.74**	0.21	**2.04**
$X_{7}$	0.64	0.03	0.05	0.00	−0.04	−0.06	−0.03	−0.01
$X_{8}$	0.88	**0.39**	**0.30**	0.26	0.28	**0.83**	0.19	**2.54**
$X_{9}$	0.80	0.05	0.06	0.07	0.15	0.34	0.09	0.64
$X_{10}$	0.30	0.00	0.10	0.10	0.18	0.48	0.17	**1.03**
$X_{11}$	0.52	0.00	0.17	0.14	0.21	0.61	0.14	**1.36**
$X_{12}$	0.48	0.00	0.07	0.06	−0.17	−0.29	−0.14	−0.50

*Note.* Bold-faced values are above the heuristic rule for that item index. MS = Molenaar–Sijtsma method; CA = correction for attenuation.

## Discussion

Methods MS, $\lambda_{6}$, and LCRC were adjusted for estimating item-score reliability. Method CA was an existing method. The simulation study showed that methods MS and CA had the smallest median bias. Method $\lambda_{6}$ estimated $\rho_{\mathrm{ii}}\prime \;$ with the smallest variability, but this method underestimated item-score reliability in all conditions, probably because it is lower bound to the reliability, rendering it highly conservative. The median bias of method LCRC across conditions was almost 0, but the method showed large variability and produced many outliers overestimating item-score reliability.

It was concluded that in the unequal α-parameters condition and in the two-dimensional condition, the methods do not estimate item-score reliability very accurately (based on median bias, IQR, and percentage of outliers). Compared with the standard condition, for unequal α-parameters, for high-discrimination items, median bias is large, variability is larger, and percentage of outliers is smaller. The same conclusion holds for the multidimensional condition. In practice, unequal α-parameters across items and multidimensionality are common, implying that $\rho_{\mathrm{ii}}\prime$ is underestimated. In the other conditions, methods MS and CA produced the smallest median bias and the smallest variability, while method $\lambda_{6}$ produced small variability but showed larger negative median bias which rendered it conservative. Method LCRC showed small median bias, but large variability.

The authors conjecture that the way the fit of the LCM is established causes the large variability, and provide some preliminary thoughts for dichotomous items. For the population probabilities $\pi_{1(i)}$ and $\pi_{1(i),1(i\prime )}$ defined earlier, let ${\hat{\pi }}_{1(i)}=\sum_{k}P(\hat{\xi }=k)P(X_{i}=1|\hat{\xi }=k)$ and ${\hat{\pi }}_{1(i),1(i\prime )}=\sum_{k}P(\hat{\xi }=k)(P[X_{i}=1|\hat{\xi }=k]{)}^{2}$ be the their latent class estimates based on sample data, and let $p_{1(i)}$ denote the sample proportion of respondents that have score 1 on item i. For dichotomous items, the item-score reliability (Equation 4) reduces to

$$\rho_{\mathrm{ii}}\prime =\frac{\pi_{1\left(i\right),1\left(i\prime \right)}-\pi_{1\left(i\right)}^{2}}{\pi_{1\left(i\right)}\left(1-\pi_{1\left(i\right)}\right)}.$$

In samples, method LCRC estimates Equation 17 by means of

$${\hat{\rho }}_{\mathrm{ii}}\prime =\frac{{\hat{\pi }}_{1\left(i\right),1\left(i\prime \right)}-p_{1\left(i\right)}^{2}}{p_{1\left(i\right)}\left(1-p_{1\left(i\right)}\right)}.$$

The fit of a LCM is based on a distance measure between ${\hat{\pi }}_{1(i)}$ and $p_{1(i)}$. However, the fit of the LCM is not directly relevant for Equation 18, because ${\hat{\pi }}_{1(i)}$ does not play a role in this equation. A more relevant fit measure for Equation 18 would be based on a distance measure between ${\hat{\pi }}_{1(i),1(i\prime )}$ and an observable quantity, but such a fit measure is unavailable. The impact of ${\hat{\pi }}_{1(i),1(i\prime )}$ not being considered in the model fit is illustrated by means of the following example. Table 5 shows the parameter estimates of LCMs with two and three classes that both produce perfect fit, that is, one can derive from the parameter estimates that for both models ${\hat{\pi }}_{1(i)}=p_{1(i)}=.68$. In addition, one can also derive from the parameter estimates that for the two-class model, ${\hat{\pi }}_{1(i),1(i\prime )}=.484$ and ${\hat{\rho }}_{\mathrm{ii}}\prime =.099$, whereas for the three-class model, ${\hat{\pi }}_{1(i),1(i\prime )}=.508$ and ${\hat{\rho }}_{\mathrm{ii}}\prime =.210$. This example shows that, although the two LCMs both show perfect fit, the resulting values of ${\hat{\rho }}_{\mathrm{ii}}\prime$ vary considerably. Hence, the variability of the LCRC estimate is larger than the fit of the LCM, and this may explain the large variability of method LCRC in the simulation study.

**Table 5.** Parameters of Latent Class Models Having Two and Three Classes.

Two-class model		Three-class model	
Class weights	Response probabilities	Class weights	Response probabilities
$P(\hat{\xi }=1)=.4$	$P(X_{i}=1|\hat{\xi }=1)=.5$	$P(\hat{\xi }=1)=.4$	$P(X_{i}=1|\hat{\xi }=1)=.5$
$P(\hat{\xi }=2)=.6$	$P(X_{i}=1|\hat{\xi }=2)=.8$	$P(\hat{\xi }=2)=.3$	$P(X_{i}=1|\hat{\xi }=2)=.6$
		$P(\hat{\xi }=3)=.3$	$P(X_{i}=1|\hat{\xi }=3)=1.0$

Values for item-score reliability ranging from .05 to .41 were used. These values are small compared with values suggested in the literature. For example, Wanous and Reichers (1996) suggested a minimally acceptable item-reliability of .70 in the context of overall job satisfaction, and Ginns and Barrie (2004) suggested values in excess of .90. It was believed that for most applications, such high values may not be realistic. In the real-data example, item-score reliability estimates ranged from <.01 to .47. Further research is required to determine realistic values of item-reliability. In this study, the range of investigated values for $\rho_{\mathrm{ii}}\prime \;$ was restricted. The item-score reliability methods’ behavior should be investigated under different conditions for a broader range of values for $\rho_{\mathrm{ii}}\prime$. This research is now under way.

## Appendix

### Coefficient Alpha

An item-score reliability coefficient based on coefficient α can be constructed as follows. Let ${\tilde{\pi }}_{x(i),y(i\prime )}^{\alpha }$ be an approximation of $\pi_{x(i),y(i\prime )}$ based on observable probabilities, such that replacing $\pi_{x(i),y(i\prime )}$ in the right-hand side of Equation 3 by ${\tilde{\pi }}_{x(i),y(i\prime )}^{\alpha }$ results in coefficient α, that is,

$$\alpha =\frac{\underset{i\neq j}{\sum \sum }\sum_{x}\sum_{y}\left[\pi_{x\left(i\right),y\left(j\right)}-\pi_{x\left(i\right)}\pi_{y\left(j\right)}\right]}{\sigma_{X}^{2}}+\frac{\sum_{i}\sum_{x}\sum_{y}\left[{\tilde{\pi }}_{x\left(i\right),y\left(i\prime \right)}^{\alpha }-\pi_{x\left(i\right)}\pi_{y\left(i\right)}\right]}{\sigma_{X}^{2}}.$$

Van der Ark et al. (2011) showed that the numerator of the ratio on the right-hand side equals

$$\sum_{i}\sum_{x}\sum_{y}\left[{\tilde{\pi }}_{x\left(i\right),y\left(i\prime \right)}^{\alpha }-\pi_{x\left(i\right)}\pi_{y\left(i\right)}\right]=Jm^{2}\bar{\pi },$$

where $\bar{\pi }$ is the mean of the $J(J-1)m^{2}$ observable terms in the numerator of the first ratio in Equation A3,

$$\bar{\pi }=\frac{\underset{i\neq j}{\sum \sum }\sum_{x}\sum_{y}\left[\pi_{x\left(i\right),y\left(j\right)}-\pi_{x\left(i\right)}\pi_{y\left(j\right)}\right]}{J\left(J-1\right)m^{2}}.$$

Hence, coefficient α equals

$$\alpha =\frac{\underset{i\neq j}{\sum \sum }\sum_{x}\sum_{y}\left[\pi_{x\left(i\right),y\left(j\right)}-\pi_{x\left(i\right)}\pi_{y\left(j\right)}\right]}{\sigma_{X}^{2}}+\frac{Jm^{2}\bar{\pi }}{\sigma_{X}^{2}}.$$

Let $w_{i}$ be an arbitrary weight with $w_{i}\geq 0$ and $\sum_{i}w_{i}=1$. Coefficient α in Equation A4 can also be written as

$$\alpha =\frac{\underset{i\neq j}{\sum \sum }\sum_{x}\sum_{y}\left[\pi_{x\left(i\right),y\left(j\right)}-\pi_{x\left(i\right)}\pi_{y\left(j\right)}\right]}{\sigma_{X}^{2}}+\frac{\sum_{i}w_{i}Jm^{2}\bar{\pi }}{\sigma_{X}^{2}}.$$

The aim of including $w_{i}$ in the definition of α is to demonstrate identifiability problems in α for item scores. Consistent with Equation 4, for an item score i, Equation A5 may be reduced to

$$\alpha_{i}=\frac{w_{i}\bar{\pi }}{\sigma_{X_{i}}^{2}}.$$

Because $w_{i}$ is arbitrary, coefficient α for item scores is unidentifiable, which makes this item-score reliability coefficient unsuited for estimating item-score reliability. Note that a natural choice would be to have $w_{i}=1$ for all i. In that case, the numerator of Equation A6 is a constant and coefficient α for item scores is completely determined by the variance of the item.

#### Coefficient $\lambda_{2}$

A line of reasoning similar to that for coefficient α can be applied to coefficient $\lambda_{2}$. Let ${\tilde{\pi }}_{x(i),y(i\prime )}^{\lambda_{2}}$ be an approximation of $\pi_{x(i),y(i\prime )}$ based on observable probabilities, such that replacing $\pi_{x(i),y(i\prime )}$ in Equation A3 by ${\tilde{\pi }}_{x(i),y(i\prime )}^{\lambda_{2}}$ results in coefficient $\lambda_{2}$; that is,

$$\lambda_{2}=\frac{\underset{i\neq j}{\sum \sum }\sum_{x}\sum_{y}\left[\pi_{x\left(i\right),y\left(j\right)}-\pi_{x\left(i\right)}\pi_{y\left(j\right)}\right]}{\sigma_{X}^{2}}+\frac{\sum_{i}\sum_{x}\sum_{y}\left[{\tilde{\pi }}_{x\left(i\right),y\left(i\prime \right)}^{\lambda_{2}}-\pi_{x\left(i\right)}\pi_{y\left(i\right)}\right]}{\sigma_{X}^{2}}.$$

Van der Ark et al. (2011) showed that

$$\alpha =\frac{\sum \sum_{i\neq j}\sum_{x}\sum_{y}\left[\pi_{x\left(i\right),y\left(j\right)}-\pi_{x\left(i\right)}\pi_{y\left(j\right)}\right]}{\sigma_{X}^{2}}+\frac{Jm^{2}\bar{\pi }}{\sigma_{X}^{2}}.$$

Hence, coefficient $\lambda_{2}$ equals

$$\lambda_{2}=\frac{\underset{i\neq j}{\sum \sum }\sum_{x}\sum_{y}\left[\pi_{x\left(i\right),y\left(j\right)}-\pi_{x\left(i\right)}\pi_{y\left(j\right)}\right]}{\sigma_{X}^{2}}+\frac{\gamma }{\sigma_{X}^{2}}.$$

Let $w_{\mathrm{ixy}}$ be an arbitrary weight with $w_{\mathrm{ixy}}\geq 0$ and $\sum_{i}\sum_{x}\sum_{y}w_{\mathrm{ixy}}=m^{2}J$. Using weights $w_{i}$, coefficient $\lambda_{2}$ in Equation A9 can also be written as

$$\lambda_{2}=\frac{\underset{i\neq j}{\sum \sum }\sum_{x}\sum_{y}\left[\pi_{x\left(i\right),y\left(j\right)}-\pi_{x\left(i\right)}\pi_{y\left(j\right)}\right]}{\sigma_{X}^{2}}+\frac{\sum_{i}w_{i}\gamma }{\sigma_{X}^{2}}.$$

Consistent with Equation 4, for an item score i, based on Equation A10, consider

$$\lambda_{2_{i}}=\frac{w_{i}\gamma }{\sigma_{X_{i}}^{2}}.$$

Similar to the item version of coefficient α, the item version of coefficient $\lambda_{2}$ is unidentifiable because $w_{i}$ can have multiple values, which renders this version of coefficient $\lambda_{2}$ not a candidate to estimate $\rho_{\mathrm{ii}\prime }$. Setting $w_{i}$ to 1 results in a coefficient that depends on the item variance, making it unsuited as a coefficient for item-score reliability.
